# Knockout of angiotensin converting enzyme-2 receptor leads to morphological aberrations in rodent olfactory centers and dysfunctions associated with sense of smell

**DOI:** 10.3389/fnins.2023.1180868

**Published:** 2023-06-19

**Authors:** Sarang Mahajan, Deepshikha Sen, Anantu Sunil, Priyadharshini Srikanth, Shruti D. Marathe, Karishma Shaw, Mahesh Sahare, Sanjeev Galande, Nixon M. Abraham

**Affiliations:** ^1^Laboratory of Neural Circuits and Behaviour (LNCB), Department of Biology, Indian Institute of Science Education and Research (IISER), Pune, Maharashtra, India; ^2^Department of Biology, Indian Institute of Science Education and Research (IISER), Pune, Maharashtra, India; ^3^Indian Institute of Science Education and Research (IISER), Kolkata, West Bengal, India; ^4^Laboratory of Chromatin Biology and Epigenetics, Department of Biology, Indian Institute of Science Education and Research (IISER), Pune, Maharashtra, India; ^5^Center of Excellence in Epigenetics, Department of Life Sciences, Shiv Nadar University, Delhi-NCR, India

**Keywords:** ACE2 receptor, gene knockout, CRISPR-Cas9, olfactory system, sensory and cognitive deficits

## Abstract

Neuronal morphological characterization and behavioral phenotyping in mouse models help dissecting neural mechanisms of brain disorders. Olfactory dysfunctions and other cognitive problems were widely reported in asymptomatic carriers and symptomatic patients infected with Severe Acute Respiratory Syndrome Coronavirus-2 (SARS-CoV-2). This led us to generate the knockout mouse model for Angiotensin Converting Enzyme-2 (ACE2) receptor, one of the molecular factors mediating SARS-CoV-2 entry to the central nervous system, using CRISPR-Cas9 based genome editing tools. ACE2 receptors and Transmembrane Serine Protease-2 (TMPRSS2) are widely expressed in the supporting (sustentacular) cells of human and rodent olfactory epithelium, however, not in the olfactory sensory neurons (OSNs). Hence, acute inflammation induced changes due to viral infection in the olfactory epithelium may explain transient changes in olfactory detectabilities. As ACE2 receptors are expressed in different olfactory centers and higher brain areas, we studied the morphological changes in the olfactory epithelium (OE) and olfactory bulb (OB) of ACE2 KO mice in comparison with wild type animals. Our results showed reduced thickness of OSN layer in the OE, and a decrease in cross-sectional area of glomeruli in the OB. Aberrations in the olfactory circuits were revealed by lowered immunoreactivity toward microtubule associated protein 2 (MAP2) in the glomerular layer of ACE2 KO mice. Further, to understand if these morphological alterations lead to compromised sensory and cognitive abilities, we performed an array of behavioral assays probing their olfactory subsystems’ performances. ACE2 KO mice exhibited slower learning of odor discriminations at the threshold levels and novel odor identification impairments. Further, ACE2 KO mice failed to memorize the pheromonal locations while trained on a multimodal task implying the aberrations of neural circuits involved in higher cognitive functions. Our results thus provide the morphological basis for the sensory and cognitive disabilities caused by the deletion of ACE2 receptors and offer a potential experimental approach to study the neural circuit mechanisms of cognitive impairments observed in long COVID.

## 1. Introduction

Angiotensin converting enzyme (ACE) 2 plays a critical role in maintaining physiological homeostasis (Donoghue et al., 2000; Hamming et al., 2004). It is widely expressed in different body systems and was identified as one of the molecular factors mediating Coronavirus disease 2019 (COVID-19) infection (Brann et al., 2020; Fodoulian et al., 2020; Hoffmann et al., 2020). Most of the mortality caused by COVID-19 infection have been reported to be due to severe respiratory problems. This was caused by malfunctioning of cardiovascular and respiratory systems (Crackower et al., 2002; Hoffmann et al., 2020). The defects associated with lung function can be a predictor for neurological impairments as well (Mahammedi et al., 2021). Since the beginning of pandemic, autopsy studies reported the presence of viral particles in multiple organ systems including the nervous system (Puelles et al., 2020). Brain imaging data further confirmed the structural abnormalities caused by the viral infection (Douaud et al., 2022). Moreover, long-lasting brain dysfunctions have become a serious challenge in post-COVID-19 conditions (Pardasani and Abraham, 2022; Bhowmik et al., 2023). Therefore, probing the mechanisms underlying these deficits using animal models is a pressing need of global health.

The binding of viral particles on ACE2 receptors leads to the creation of a fusion pore that allows viral entry into the host cells. This is assisted by the priming of spike protein by host cell transmembrane protease, serine 2 (TMPRSS2) (Shang et al., 2020; Jackson et al., 2022). Although the routes of viral entry to the central nervous system (CNS) and the neurotropism of Severe Acute Respiratory Syndrome Coronavirus 2 (SARS-CoV-2) remain as debated topics, various cellular factors mediating virus entry are expressed in neuronal and non-neuronal cells in the brain (Pardasani and Abraham, 2022). ACE2 receptors are expressed in the non-neuronal supporting (sustentacular) cells of olfactory epithelium. This explains the prevalent olfactory deficits caused by different strains of SARS-CoV-2 (Bhattacharjee et al., 2020; Whitcroft and Hummel, 2020; Iravani et al., 2022), supported by the observations on presence of viral RNA and protein in the nasopharynx (Meinhardt et al., 2021). However, some studies did not find the presence of viral particles in the olfactory sensory neurons (OSNs) and olfactory bulb (OB), leading to the debate on the neurotropism of SARS-CoV-2 (Khan et al., 2021). Another receptor type that facilitates virus entry, Neuropilin-1 (NRP1) is expressed in the neurons, olfactory epithelial cells, and endothelial cells etc. (Cantuti-Castelvetri et al., 2020; Kyrou et al., 2021). Despite the above-mentioned evidence on molecular factors, studies dissecting the neural basis of olfactory and cognitive deficits using animal models are scarce.

Single cell sequencing studies of mouse olfactory epithelium revealed the expression of ACE2 and TMPRSS2 in the sustentacular cells, however not in the OSNs (Brann et al., 2020; Fodoulian et al., 2020; Hoffmann et al., 2020). Transnasal infusion of viral particles in Golden Syrian Hamsters provided the evidence for neuronal invasion. Both neuronal and non-neuronal cell deaths were observed during the post-infection period (De Melo et al., 2021). As genetic approaches mimicking viral infection can provide stable readouts, we decided to generate ACE2 receptor knockout using CRISPR-Cas9 genome editing tools. The CRISPR-Cas9 technique has been successfully used in mouse and other mammalian species to generate genetically modified animals (Shao et al., 2014; Kang et al., 2019). The deletion of ACE2 gene was ensured by targeting the crucial translational start site of the exon 2 and verified by sequencing. On generating ACE2 KO mice, we carried out the morphological studies and the behavioral phenotyping focusing on the functioning of olfactory system. As olfactory problems of varying severity including hyposmia, anosmia and parosmia are observed during and post-COVID conditions, we used well-established and sensitive behavioral assays (Abraham et al., 2004, 2010, 2014; Bhattacharjee et al., 2019; Pardasani et al., 2021). The reduced thickness of OSN layer and the lowered MAP2 immunoreactivity in the glomerular region explained various olfactory problems including the detection, and discrimination deficits at the threshold levels and the lowered novel odor identification abilities. Further, ACE2 KO mice showed compromised pheromone location learning, which involved more than one sensory modality. Hence, our experimental approach would facilitate probing the neural mechanisms of long-COVID complications.

## 2. Materials and methods

### 2.1. Maintenance of animals

A total of 119 C57BL/6 J and ACE2 KO male and female adult mice were used for all of the experiments in this study. The mice were between 6 to 8 weeks old at the beginning of the experiment. 12-h light/dark cycle was maintained and mice were grouped in individually ventilated cages in a temperature- and humidity-controlled animal facility. Mice had unlimited access to food, but were subjected to a water restriction schedule meant to keep them at >80% of their baseline body weight during Go/No-Go behavioral training. The schedule of water restrictions was never longer than 12 h. All animal care and procedures were in accordance with the Institutional Animal Ethics Committee (IAEC) at IISER Pune and the Committee for the Purpose of Control and Supervision of Experiments on Animals (CPCSEA), Government of India.

### 2.2. Generation of ACE2 KO mouse model

**Figure 1:**
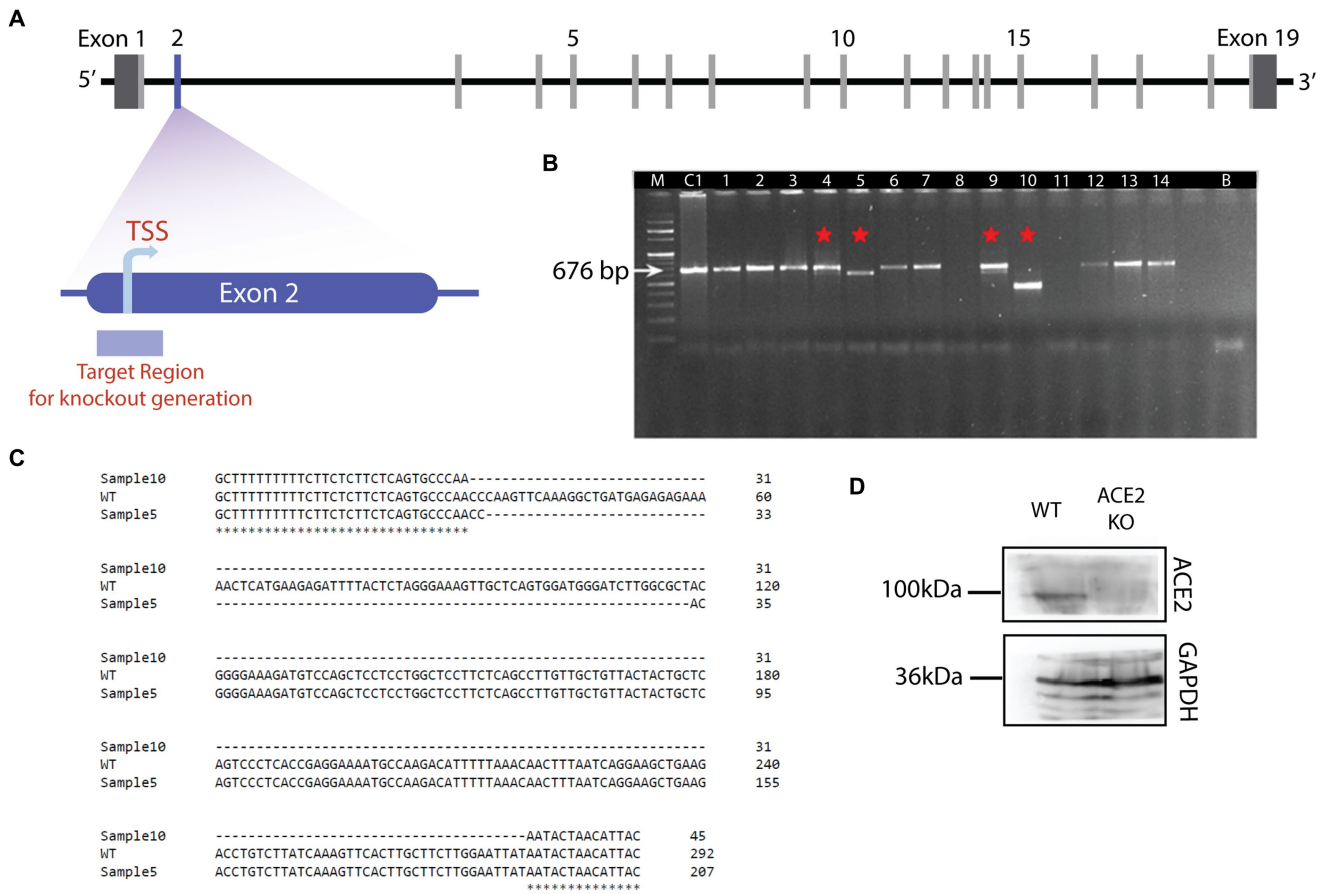
Generation of ACE2 knockout mouse model. **(A)** Schematic of the genetic structure of the ACE2 gene. ACE2 gene harbors 19 exons with the translation start site (TSS) in the exon 2. TSS is the target region to create the knockout using CRISPR-Cas9 mediated genome editing. **(B)** Agarose gel electrophoresis image of DNA samples isolated from the potential ACE2 KO animals. Sample C is the control (C57BL/6 J) genomic DNA (676 bp), Sample B is used as a negative control without any genomic DNA, and samples 1 to 14 are DNAs with genome editing. Samples 4, 5, 9, and 10 revealed the deletions and are depicted by red asterisk on the gel. Sample 10 showed the maximum deletion, hence selected for further breeding. **(C)** Sequence alignment showing deletions in samples 5 and 10. For the sample 5, 84 bp deletion was observed, whereas for sample 10, a 246 bp deletion occurred in the target region. These results confirmed the deletion of the ACE2 receptor gene in sample 10. **(D)** Western blot showing the expression of ACE2 protein in the brain of ACE2 KO and WT animals. The band corresponding to ACE2 protein was observed for WT animals, whereas in ACE2 KO animals, ACE2 expression was undetectable. Band corresponding to GAPDH protein is observed in brain lysates from both animals.

Using CRISPR-Cas9 gene targeting technology, we generated a knockout mouse model for ACE2. The knockout was created by specifically targeting the translation start site (TSS), which lies in the exon 2 of the ACE2 gene (Figure 1A). Guide RNAs for the 5*′* and 3*′* ends of the targeted region were chosen to generate the knockout (ATCAGCCTTTGAACTTGGGT; ATCAAAGTTCACTTGCTTCT). SgRNAs were designed using the CRISPOR online tool^1^ and synthesized by Sigma Aldrich.

### 2.3. Microinjection of one-cell embryos

C57BL/6 J mice at 3–4 weeks of age were superovulated by intraperitoneal injection of 5 IU pregnant mare serum gonadotropin (PMSG), followed by injection of 5 IU human chorionic gonadotropin (Sigma Aldrich) after 48 h. Mouse zygotes were obtained by mating C57BL/6 J stud males with superovulated C57BL/6 J females. One-cell stage fertilized mouse embryos were injected with CRISPR components mixed in microinjection buffer. The final concentrations of Cas9 protein and sgRNA were 50 ng/μL and 25 ng/μL, respectively. The fertilized one cell embryos were isolated from superovulated female mice. Microinjection of the mixture was performed into pronuclei of fertilized eggs using FemtoJet 4i microinjector with manipulator (Eppendorf) attached to IX83 microscope (Olympus). The injected embryos were transferred into the oviduct of pseudo-pregnant females to allow further development. Microinjections and mouse transgenesis experiments were performed as described previously (Harms et al., 2014).

The resulting pups were genotyped for founder screening. The primers used for PCR were (ACE2-F1: 5*′*- ACCCTCCTCCTCCAGTG TAT −3*′* and ACE2-R1: 5*′*- AGGCAGTCACTCATCCTCAC -3*′*). PCR was conducted using the following conditions: Initial denaturation at 95°C for 4 min, 36 cycles with denaturation at 94°C (30 s), annealing at 60°C (30 s), and extension at 68°C (1 min). Final extension was performed at 68°C for 5 min. The deletion of the ACE2 gene’s target site in mice was identified and confirmed by using polymerase chain reaction (PCR) and gene sequencing. The sequence confirmed founders were backcrossed to wild-type C57BL/6 J mice for two consecutive generations and the founder line was established. Animals with confirmed ACE2 receptor gene knockout were used for breeding.

### 2.4. Western blotting

For Western Blotting (WB), 6–10 weeks old wild type and ACE2 KO animals were used. Mouse brains were dissected and stored at −80°C. Whole brain lysates were prepared in RIPA buffer supplemented with cOmplete protease inhibitor (Roche Cat # 04693116001). Protein estimation was performed using Pierce BCA protein assay kit (Thermofisher Cat # 23225). Fifteen μg of the sample was loaded in each well of 12% acrylamide gel and SDS–polyacrylamide gel electrophoresis (PAGE) was performed. The proteins were then transferred to Immobilon-P PVDF membranes (Millipore Cat # IPVH00010). Blocking was performed with 5% milk/Tris-buffered saline–Tween 20 for 1 h at room temperature. The membranes were probed with primary anti-ACE2 antibody (Abcam Cat # 15348) and anti-GAPDH (Sigma Cat # G9545) at 1:1000 and 1:5000 dilutions, respectively at 4° C for 16 h. The secondary antibody used was peroxidase-conjugated AffiniPure Goat anti-rabbit IgG (Jackson ImmunoResearch Cat # 111–035-003) at 1:5000 dilution for 1 h at room temperature. Bound antibody was detected using Clarity ECL Western Blotting Substrate (BioRad Cat # 1705061) with the image digitally captured using an ImageQuant LAS 4000 imager.

### 2.5. Hematoxylin and eosin staining of olfactory epithelium

**Figure 2:**
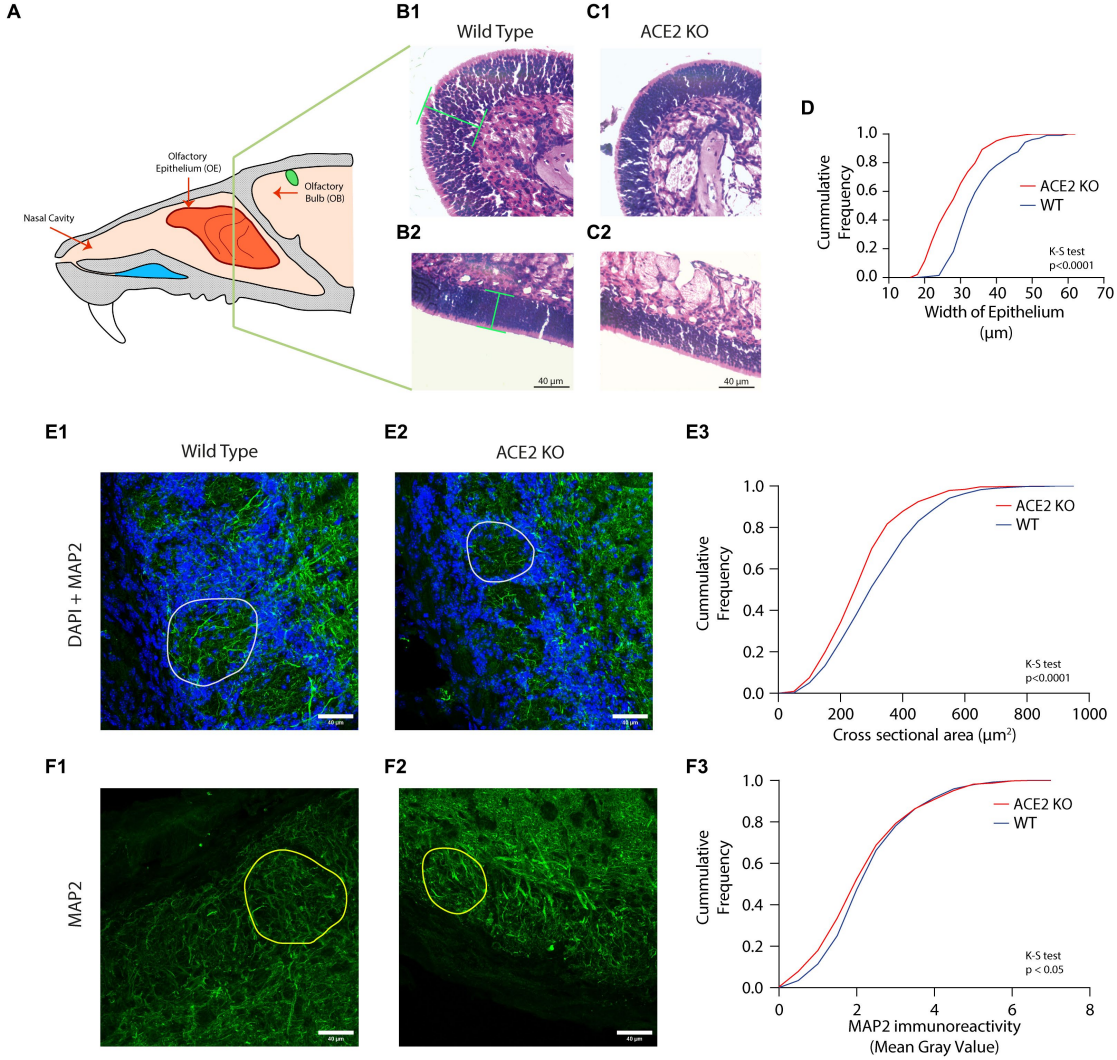
Morphological aberrations in the olfactory epithelium (OE) and olfactory bulb (OB) of ACE2 KO animals. **(A)** Schematic representation of the mouse olfactory system. The olfactory epithelium (OE) is present in the posterior region of the nasal cavity and harbors olfactory sensory neurons (OSNs). These OSNs express odor receptors. The signal from the OSNs is then transduced to the olfactory bulb (OB). **(B,C)** Representative images of different regions of OE stained with Hematoxylin and Eosin for WT **(B1,B2)** and ACE2 KO **(C1,C2)** animals, respectively. Green lines in the panel **B1** and **B2** represent the width of the epithelium. **(D)** Cumulative frequency distribution of epithelium width for ACE2 KO and WT animals. The width of epithelium for WT was 35.39 ± 0.5294 μm, ACE2 KO: 28.67 ± 0.4408, K-S test, *p* < 0.0001, number of animals: n_WT_ = 4 and n_ACE2 KO_ = 4, number of region of interests (ROI): WT = 211, ACE2 KO = 232. **(E)** Representative images of the glomerular layer of the OB stained with DAPI and MAP2 from WT **(E1)** and ACE2 KO animals **(E2)**. DAPI is visualized with blue color, whereas MAP2 is visualized with green color. **(E3)** Cumulative frequency distributions of cross-sectional area of the glomeruli pooled for WT and ACE2 KO animals. The cross-sectional area for WT was 335.6 ± 6.444 μm^2^, for ACE2 KO was 283.1± 5.191 μm^2^ (K-S test, *p* < 0.0001, number of animals: n_WT_ = 3 and n_ACE2 KO_ = 3, number of glomeruli: WT = 496, ACE2 KO = 558). **(F)** Representative images of the glomerular layer of the OB stained with MAP2 (green color) for WT **(F1)** and ACE2 KO animals **(F2)**. Yellow colored circles in the images represent individual glomeruli. **(F3)** Cumulative frequency distributions of MAP2 immunoreactivity measured using mean intensity for WT and ACE2 KO animals. The MAP2 immunoreactivity for WT was 2.490 ± 0.0499, for ACE2 KO was 2.357 ± 0.0517 (K-S test, *p* = 0.0349, number of animals: n_WT_ = 3 and n_ACE2 KO_ = 3, number of glomeruli: WT = 496, ACE2 KO = 558).

The mice were initially perfused with 1 × Phosphate Buffer Saline (PBS) followed by 4% paraformaldehyde. The animal was decapitated, and the nasal cavity was dissected. After that, tissue was kept for a week in a 10% Ethylenediamine tetraacetic acid (EDTA) solution to decalcify the bones that surround the nasal cavity. After the removal of tissue from the EDTA solution, the nasal cavity was extracted by delicately removing the surrounding bones. Following the dissection of the nasal cavity, it was embedded in the paraffin wax. Briefly, the nasal cavity was kept in 60% isopropanol (90 min × 3 times), 80% isopropanol (45 min × 2 times), 90% isopropanol (30 min × 1 time followed by 15 min × 1 time), and then in xylene (15 min × 3 times). The dehydrated nasal cavity was then placed over previously melted paraffin wax and incubated overnight at 62°C in an oven. Following that tissue was embedded in a wax block and was incubated at −20°C overnight. Using microtome (RM2235, Leica Biosystems), 5–8 μm sections were obtained and were transferred to the poly-L-lysine coated slides. The slides were then incubated in a hybridization oven overnight at 62°C. Then, the slides were kept in xylene (5 min × 2 times) followed by varying concentrations of ethanol: 100, 90, 70, and 50% for 3 min each. The slides were then kept in distilled water for 3 min. The slides were removed from the water and left to air dry. A napkin was used to wipe away any excess water surrounding the tissue. Slides were positioned on the rails of the humidifying chamber, and a drop of hematoxylin was applied to the tissue and slides were left undisturbed for 15 min. The excess hematoxylin stain was removed with distilled water, and the slides were rinsed under running water for 15 min. The slides were then submerged for 10 s in 80% ethanol containing 1% Hydrochloric acid (HCl). To each tissue section, a drop of eosin was added. After 30 s of eosin application, slides were transferred to 70% ethanol for 1 min, 90% ethanol for 1 min, and 100% ethanol for 1 min. The slides were taken out of the ethanol and let to air dry. Following that slides were kept in xylene (15 min × 2 times). The slides were taken out, and before the slides dried fully, excess xylene was removed from the corners of the slides using a napkin and a drop of DPX medium was applied to the sections. The slides were mounted with a cover slip and edges of the cover slip were sealed. A brightfield microscope (BX43, Olympus) was next used to examine the sections and capture the images. We selected similar regions in the medio-lateral and anterio-posterior axes on the nasal turbinates for both WT and KO mice. While calculating the cumulative distributions, 3–4 regions of interest (ROIs) from 18 to 24 sections per animal were selected to cover the nasal epithelium (Figure 2D). In addition, we have measured the epithelial thickness in different areas near to the septum (henceforth named as septal areas, dorsomedial and middle meatus areas) and other turbinate regions toward the lateral side (ethmoturbinate areas) in each section, with similar numbers from both locations (Supplementary Figure S1). The thickness of OE was measured across OSN layer at different locations (as marked in Figures 2B1,B2).

### 2.6. Immunohistochemistry using MAP2

ACE2 KO and WT animals were perfused, and their olfactory bulbs (OB) were dissected. The dissected brain was kept in 4% paraformaldehyde for 24 h before keeping it in 30% sucrose for 1 day for cryoprotection. For Microtubule associated protein 2 (MAP2) staining, 50 μm thick sections were obtained using cryotome (CM1950, Leica Biosystems) and one section was selected for every five sections. Sections were washed three times in tris-buffered saline (1 × TBS) for 15 min on a rocker at 15 rpm. The permeabilization and blocking were done using TBST (0.2% Triton X-100 in 1 × TBS) with 5% bovine serum albumin (BSA) and 7.5% normal goat serum (NGS) for 1 h at room temperature. The sections were then incubated overnight at 4°C with primary antibody diluted in the respective blocking buffers. Chicken anti-MAP2 (Abcam, ab92434, 1:3000) was used as the primary antibody. Sections were then washed with 1 × TBS (10 min × 3 times) and incubated for 1 h at room temperature with secondary antibody diluted in TBST. Alexa Fluor 647-conjugated donkey anti-chicken IgG (Jackson ImmunoResearch, 703–605-155,1:500) was used as the secondary antibody. The sections were then given 1 × TBS (10 min × 3 times) washes and were incubated with DAPI (Sigma, 1:500) in 1 × TBS for 10 min. Following that, sections were washed once with 1 × TBS (10 min) and then mounted onto slides with glass coverslips using a mounting medium. The mounted sections were imaged using a confocal microscope (SP8, Leica Biosystems). All image acquisition parameters, such as the objective (40X oil immersion), zoom (1.0), pinhole diameter (1 AU), pixel format (1024×1024), laser intensity (~5%), and scanned thickness of ~20 μm (1 μm step size), were kept constant during the imaging.

The area of the glomeruli and the MAP2 immunoreactivity were quantified from maximum intensity projection (MIP) images containing two channels: DAPI and MAP2 (Figure 2E, Blue and Green color, respectively). In the MIP images, each glomerulus representing an ROI was selected using the freehand selection tool in ImageJ/Fiji. The area and mean gray value (MAP2 channel) were measured for each glomerulus. To rule out any sampling bias that may result from the size distribution of glomeruli in the whole OB, we took every fifth coronal section of the OB (50 μm sections, 12–14 sections of both OBs per mouse) covering the entire anterior–posterior axis. Similar numbers of ROIs were selected in mediolateral and dorsoventral axes in both WT and ACE2 KO mice.

### 2.7. Go/no-go odor discrimination

#### 2.7.1. Odors

For Go/No-Go discrimination task, following odors were used: Methyl Benzoate (MB), Limonene (+) (Li), Amyl acetate (AA), and Ethyl butyrate (EB). The odors were diluted in mineral oil (MO) and different dilution of the odors were used (10^−4^ to 10^−10^ percent volume in MO). All odors had a purity level of above 99% and were purchased from Sigma-Aldrich, and the mineral oil was obtained from Oswal Pharmaceuticals in Pune, Maharashtra, India.

#### 2.7.2. Odor pairs

Different sets of animals were trained to differentiate the following: Li vs. MO (10^−10^, 10^−9^, and 10^−8^% v/v), MB vs. MO (10^−10^, 10^−8^, and 10^−6^% v/v), and complex binary mixture of AA vs. EB [AA(60%) + EB(40%) vs. EB(60%) + AA(40%)] (10^−8^, 10^−6^, and 10^−4^% v/v).

#### 2.7.3. Apparatus

For olfactory-based discrimination experiments, eight-channel olfactometer with custom modifications was used (Knosys). The apparatus consisted of an operant chamber where the animal was kept during the behavioral test. The operant chamber has a combined odor sampling and reward port on one side that is guarded by an IR beam. This allows for a tight association between odor presentation in a trial and the reward. A trial is initiated when the animal breaks the IR beam by poking its head into the sampling port. The odor valve connected to a flowmeter opens as the trial initiates and it controls the onset and flow of odor stream (airflow rate of 2 Liters per min). After 500 ms of odor valve opening, a final valve (diversion valve) that lies near to the sampling port, opens and the stimulus is delivered to the animal. The precise onset of the stimulation was ensured by a system of these solenoid valves that are controlled by custom written program in IGOR. The S+ (rewarded) or the S- (non-rewarded) odors were presented through a set of different valves.

#### 2.7.4. Task-habituation phase

The animals were subjected to task habituation training three to 4 days after the beginning of the water deprivation schedule. Standard operant conditioning approaches were used to train the animals. The task habituation phase was performed for the animals to get acclimatized to the operant chamber, location of reward and sampling port, lick tube, sounds of the valves, and procedural aspects of the instrument. The task habituation consisted of nine phases (Phase 0–8). In the Phase 0, animal received water reward (3–5 μL) simply by breaking the IR beam. This enabled the animals to locate the reward port and the water delivery tube. In the following phase, the animals were given water only when they made at least one lick. For the subsequent stages of this task-habituation training, the complexity level of the task increased gradually, and animals had to lick on the tube in order to receive the water reward. In the late stages of this phase, odor valve was introduced and animal received the odor stimulus for 2 s, wherein animal has to respond and lick to receive the reward. All animals finished the task habituation phase in three to four sessions of 30 min.

#### 2.7.5. Discrimination training phase

The odor-based discrimination tasks were performed using a Go/No-Go behavioral paradigm (Abraham et al., 2004). The mouse initiated a trial by breaking the IR beam that was guarding the sampling port. This enabled the opening of one of the solenoid valves, followed by the opening of a three-way diversion valve after 500 milliseconds. After diversion valve is opened the stimulus is presented to the animal for a 2 s duration. The use of a diversion valve reduced the period between the onset of the stimulus and the first contact with the animal. To obtain a reward, the animal has to meet the required reward criteria based on the reward contingency of the stimulus [Rewarded (S+)/ Non-Rewarded (S-)].

The time that was provided for animals to respond overlapped with the stimulus duration. The response time was virtually divided into four equal bins, i.e., for a response/stimulus duration of 2 s, divided into four 500 ms bins. Animals required to register a lick in at least three out of these four bins for a S+ trial to be considered correct. For a successful S+ trial, a water reward of 3–4 μL was given to the animal after the stimulus ended [Reward Criteria: Animal needs to register a lick in at least three out of the four bins]. For an S- trial to be correct, animal was only allowed to lick for at most two bins. There was no punishment or reward for an incorrect or correct S- trial, respectively. Before the next trial could be initiated, a 5-s inter-trial interval (ITI) was kept. There were no rules requiring the mouse to smell the odor for a certain amount of time before making a choice and to prohibit licking prior to the odor. The mice received stimuli in blocks of 20 trials. Ten S+ trials and ten S- trials were present in each of these blocks. Within a block, the S+ and S- trials were pseudorandomized in order to prevent the delivery of more than two consecutive stimuli with the same reward condition. The preference for a particular stimulus was prevented by balancing the S+ and S- stimuli for a group of animals (for instance, in a group of 8 animals, 4 mice receive one stimulus as S+ while the other 4 animals receive another stimulus as S+). The animals were adequately motivated to finish 200–300 trials in a day, spaced out over 1–2 (30–40 min) sessions. Animal’s motivation was measured using different instrumental readouts, including licking probability and inter-trial interval. The training session was terminated after the animal stopped licking for the rewarded trials. The data was collected using a custom-written software in IGOR-PRO that was compatible with the MCC-CIO-DIO 48 data acquisition card.

#### 2.7.6. Behavioral readouts

##### 2.7.6.1. Learning curve

The learning curve measures the performance as the percentage of correct responses during the training. Each point on the learning curve indicates the average accuracy of 100 trails [50 S+ and 50 S-] across all animals.

##### 2.7.6.2. D-prime (d’)

Hit (correct S+) and false alarm (incorrect S-) probabilities were computed for d’ over an average of 100 trials. The probabilities were used to calculate the z-score. d’ was calculated as z(hit)-z(false alarm) per 100 trials.

##### 2.7.6.3. Discrimination time

The licking behavior of each mouse was monitored to assess the discrimination time. Animals’ licking behavior was recorded with high temporal resolution and analyzed in time bins of 20 ms. The licking behavior changed as a result of learning and was considerably different between the early and late stages of learning. During the early phase of learning, when the animals were not able to discriminate the S+ and S- stimuli, they licked for both the stimuli. As a result, during the initial training phase, the animal’s lick responses to S+ and S- stimuli were comparable. But as soon as they were able to differentiate between two stimuli, they began to selectively lick for S+ trials and avoid licking for S- trials, which caused a divergence in the lick responses between two stimuli. The statistical comparison of the lick responses between the S+ (150 trials) and S- (150 trials) trials was performed using one-tailed t-test. The t-test was performed for each time bin of the lick pattern between S+ and S- trials. This comparison yields a value of p curve as a function of time. In the value of p curve, the last time point where the value of p is <0.05 is taken as the discrimination time. The discrimination time was measured task wise, i.e., for 300 trials.

##### 2.7.6.4. Area under the curve (AUC)

The area under the curve (AUC) was also used to calculate the discrimination index of the animals. For AUC calculations, the lick probabilities for S+ and S- trials were used. The discrimination index was calculated as: AUC = (AUC_S+_ − AUC_S-_)/AUC_S+_.

### 2.8. Novel odor discrimination

To further compare the novel odor discrimination abilities of ACE2 KO mice with WT mice, a previously published olfactory habituation/dishabituation paradigm with slight modifications was adopted (Tillerson et al., 2006; Lehmkuhl et al., 2014). Before the experiment began, animals were kept in the experimental cage for 5 min for cage habituation. 50 μL of distilled water or odor (diluted to 1% in mineral oil) was applied on a piece of Whatman filter paper and kept inside separate, identical boxes at the two ends of a cage. For the first trial, the box containing water was placed on one side of the cage, and the box containing the odor (cineole or eugenol) was placed on the opposite side. One trial continued for 3 min during which the behavior of animals was recorded. The boxes were removed from the cage after the trial finished. Inter-trial interval of 15 min were provided between the trials. In order to prevent any location-based bias of the animal, the cage was rotated around 180° between each trial. Only one odor was used for the first five trials to ensure the odor habituation, following which, on the 6^th^ trial, this odor was replaced with a novel odor, e.g., if eugenol was used in the first five trials (habituated odor), it was replaced with cineole (novel odor) in the 6^th^ trial. For each trial the behavior of animal was videotaped and the trial videos were analyzed using EthoVision software. The amount of time the animal spent sampling a particular box was determined by how long its nose tip was inside a region around 2 cm from the perimeter of the box. These areas were chosen as the zones in the software, while the entire cage served as the arena. The cage’s length and width were used to calibrate the arena, and a sample rate of 30.00 samples per second was used. Dynamic subtraction was used to identify the mice’s nose, center, and base of tail at a dark contrast of 50–60.

### 2.9. Pheromone detection

An open field pheromone detection experiment was used to examine the pheromone detecting capacities of ACE2 KO and wild type females. Before the experiment began, all females had attained sexual maturity. The behavioral apparatus used to assess pheromonal detectabilities comprised of a chamber with dimensions of 60 cm x 45 cm. Since non-volatile odorants are found in male-soiled bedding, and volatile odorants are found in urine, the test was conducted by placing a petri dish filled with male soiled bedding and urine (~100 μL) at the center of the arena. Females were kept in the chamber for 10 min and were allowed to freely roam and explore the arena. A camera was used to record the animals’ movements, and EthoVision software was used to track them. The amount of time the females spent in the vicinity of the petri dish was used to quantify the sampling behavior.

### 2.10. Multimodal pheromonal learning

#### 2.10.1. Apparatus

Pheromone preference and odor association abilities in mice were tested using the multimodal pheromonal learning paradigm established in our lab (Pardasani et al., 2021). For this experiment, the same groups of females that were used for the pheromone detection experiment were used. The apparatus comprised of an arena with dimensions of 60 cm x 30 cm x 15 cm (length x width x height). The entire arena was divided into three spaced zones having equal areas with the help of two sliding partitions. Opening both the partitions allowed the females to explore the entire arena, whereas closing them allowed us to restrict the females in specific areas. At the opposite extremities of the arena, two 10 cm x 10 cm x 15 cm compartments with removable plates were positioned. In each chamber, a 55 mm petri dish held 100 μL of either water (the neutral stimulus in chamber 2) or urine (the attractive pheromonal stimulus in chamber 1) was kept. These chambers were guarded by the lids having orifices with different diameters (5 mm and 10 mm). Due to this, the animal was restricted to sample the volatiles coming from the chamber’s front side through the holes and they were able to associate the diameters with the volatile cues. To mitigate any bias toward the diameters of the holes, animals were counterbalanced for the association between the volatile cues and the different orifice sizes.

#### 2.10.2. Paradigm

The experimental design included a 4-day initial testing phase, a 15-day training phase, and memory tests on the 15th day after the training. The purpose of the initial testing phase was to determine whether female mice have an intrinsic preference for the zones (zone 1 containing the volatile and non-volatile pheromones from male mice & zone 2 containing neutral stimuli, water) During the early testing and training phases, the equipment was rotated by 180° every day to eliminate any directional bias toward a specific zone. Following the initial testing phase, 15 days of training was performed. During the training phase, each day, the animal was only allowed in one of the zones for 15 min (alternating between the two zones after every 5 min, 3 times). Fifteen days after the end of training phase, memory test was performed. To test the memory, all volatile and non-volatile pheromonal stimuli (urine and soiled bedding with non-volatile pheromonal traces) and neutral water stimuli were removed from the chambers of the apparatus while leaving the plates with specific diameter orifices undisturbed. Using the EthoVision program, the amount of time spent in each zone, particularly in front of chambers 1 and 2, was quantified. Animal tracks were visualized and time spent was calculated using EthoVision’s nose point feature, which is used to track animals. The number of active attempts on the plates guarding chambers 1 and 2 was manually scored by counting each nose poke through the plate as one attempt. Memory index was calculated for both the time spent and number of active attempts as: Memory Index = (Time spent or No. of active attempts in pheromone zone–Time spent or No. of active attempts in neutral stimulus zone)/ Time spent or No. of active attempts in neutral stimulus zone.

### 2.11. Behavioral tests for stress, anxiety, and motor control

Different tests were conducted to study the exploration, anxiety-like, and depression related behaviors and motor control of the animals. These tests were conducted in the following order:

#### 2.11.1. Open field test

The set-up consisted of a pseudo home cage where mice belonging to the same cage were housed for about 15 min. The dimension of the cage was 42 cm x 26 cm x 18 cm and the top of the cage was covered with a grill. In order to prevent any initial hyperactivity, the animals were acclimated in this cage. During the test, a single mouse was permitted to pass from the cage into the main arena (60 cm x 45 cm) through a little opening for 10 min. A camera mounted on a tripod stand captured the exploratory behavior of the animal. Using EthoVision tracking software, the total distance traveled, time spent in the center of the arena, latency to the center, and total time spent in the four corners were calculated.

#### 2.11.2. Elevated plus maze test

For EPM test, an elevated plus maze which was raised 50 cm above the ground was used. The apparatus constituted closed and open arms. The arms of the maze were 5 cm wide and 55 cm long. The closed arms consisted of walls that were 15 cm high. The middle zone at the junction of the four arms had a dimension of 5 cm x 5 cm. To initiate a trial, the animal was placed on this junction facing the open arm. The trials lasted for 5 min and animals were free to explore the EPM during this time. For quantitative analysis, the time spent in open vs. closed arms and the number of entries into the open and closed arms were calculated.

#### 2.11.3. Tail suspension test

For TST, mouse was suspended by its tail using a 15 cm piece of tape attached to a horizontal rod at a height of 40 cm from the ground. Each trial lasted for 6 min, following which the animals were removed from the apparatus. For analysis, time spent mobile, where the animal tries to escape, and the time spent immobile were quantified manually with a resolution of 1 s.

#### 2.11.4. Forced swim test

FST was performed using an acrylic cylinder of 15 cm diameter and 30 cm height that was filled with water (12 cm height). The mouse was placed in the water for 6 min. To prevent hypothermia after the experiment was finished, the mouse was placed in a cage covered with dry tissue which was kept on a heating pad for 15 min. The animal was then transferred to its native cage. Behavior of the animal was classified between the time spent mobile and immobile. The mobility and immobility were scored manually with a resolution of 1 s. Across ACE2 KO and wild type groups, time spent immobile was compared.

#### 2.11.5. Rotarod test

The rotarod test was performed to evaluate the balance and motor coordination. The animals were placed on a rotating rod that rotates at a speed ranging from 1 to 4 revolutions per minute. The test was completed when the mouse fell off the rod and landed on the sponge bed that was kept at the base of the apparatus. Parameters such as total time spent by the animals on the rod and the distance traveled by them were quantified and compared between the two groups.

### 2.12. Statistical analysis

GraphPad Prism 9, Microsoft Excel, and Python were used for all data and statistical analyzes in this study. For image analysis ImageJ/Fiji was used. The data is presented as cumulative distributions and Mean ± SEM. To determine the *p*-values and test for statistical significance, we used the Kolmogorov–Smirnov test (K-S test), student’s *t*-test (Normally distributed data determined using Shapiro–Wilk test), Mann–Whitney test (Non-normally distributed data), one-way and two-way ANOVA, and associated post-hoc tests.

## 3. Results

### 3.1. Generation of ACE2 KO mice using CRISPR-Cas9 genome editing tools

The olfactory system of rodents is an attractive model to study the circuit mechanisms of many brain dysfunctions. The well-mapped anatomical organization, the ease of accessibility of different olfactory centers, and olfaction being the dominant sensory modality of rodents make it an efficient tool to modulate circuit functions which give rise to specific behavioral phenotypes mimicking brain disorders. Since the beginning of pandemic, olfactory system remained as the most studied sensory system due to prevalent olfactory and cognitive dysfunctions caused by SARS CoV-2 infection. As ACE2 receptor was one of the molecular factors mediating the virus entry (Klingenstein et al., 2020), ACE2 KO mouse model was generated using CRISPR-Cas9 by deleting the translation start site of the exon 2 of the ACE2 gene (Figure 1A). Guide RNAs for 5*′* and 3*′* end of the targeted region were chosen to generate the knockout. The mouse zygote was microinjected with the transcribed gRNA/Cas9 mRNAs, after the vectors that target ACE2 gene deletion were constructed using the guide RNAs. For confirmation of the deletion, the genomic DNA of the F0 mice was subjected to the PCR and was visualized using agarose gel electrophoresis. A shorter PCR product of the expected size was visible on the agarose gel. Visualization of the agarose gel revealed deletions in samples 4, 5, 9, and 10 with sample 10 showing the maximum deletion (band shown in sample 10 of Figure 1B). The deletions were further confirmed by performing the sequencing for samples 5 and 10 in which 84 bp and 246 bp deletions were observed, respectively, (Figure 1C). F0 female mouse (sample 10) was further crossed with C57 BL6 males and the progenies were then backcrossed for three generations to obtain enough number of homozygous KO animals. The genotype of experimental mice were further confirmed by western blotting to check the ACE2 protein levels in the brains of ACE2 KO and WT animals. In contrast to WT animals, which showed prominent bands for ACE2, western blot analysis did not reveal any band corresponding to ACE2 protein in the ACE2 KO animals. Bands for GAPDH, which acted as an internal control, were observed in both groups of animals (Figure 1D), thereby confirming the absence of ACE2 proteins in the brains of animals used in the experiments. These results therefore confirm the successful generation of the ACE2 KO mouse model. Further, morphological phenotypes of these mice were assessed using microscopic techniques and the behavioral phenotypes were studied using various assays.

### 3.2. ACE2 KO animals exhibit morphological alterations in the olfactory epithelium and olfactory bulb

Although there are mouse models available to study underlying mechanisms of brain dysfunctions caused by SARS CoV-2 infection, a detailed characterization of sensory as well as cognitive deficits using precise behavioral assays are lacking to date. In the olfactory system of rodents, ACE2 receptors are primarily found in supporting sustentacular cells of the olfactory epithelium (Bilinska et al., 2020; Butowt and Bilinska, 2020; Lechien et al., 2021). Due to the protective and supporting nature of sustentacular cells, the viral infection resulting in the internalization of receptors can cause neuroinflammatory changes leading to gradual decaying of OSN functions. To investigate the effect of ACE2 receptor knockout on the morphology of OSNs, we first quantified the OSN layer thickness and compared it to that of wildtype (WT) mice (Figure 2A). Coronal sections of the OE stained with Hematoxylin and Eosin were used to measure the OE thickness, which was assessed as the perpendicular distance from the basal membrane. For ACE2 KO, 232 regions of interest (ROIs), and for WT mice, 211 ROIs were analyzed along anterior–posterior and mediolateral axes for four mice in each group. The cumulative distributions of epithelium width measurements reveal smaller OE thickness in ACE2 knockout mice in comparison to that of the WT animals, indicating the role of supporting cells in maintaining the morphology of the OE (Figures 2B–D, WT: 35.39 ± 0.5294 μm, ACE2 KO: 28.67 ± 0.4408, K-S test, *p* < 0.0001). In addition, we measured the epithelial thickness in different areas near to the septum (henceforth named as septal areas, dorsomedial and middle meatus areas) and other turbinate regions toward lateral side (ethmoturbinate areas) in each section, with similar numbers from both locations. On analyzing these areas separately, we observed lower thickness of olfactory epithelium in ACE2 KO animals in both locations, thereby confirming that reduction in epithelium thickness is independent of the location on the turbinates (Supplementary Figure S1, ACE2 KO vs. WT: K-S test, *p* < 0.0001 for both septal and ethmoturbinate areas).

The OSNs project to glomeruli of OB in a receptor specific manner. In the glomeruli, these OSNs makes synapses with the Mitral/Tufted cells, which are the output neurons of the OB. Since the morphological characteristics of OB glomeruli are dependent on the axonal inputs of OSNs (Potter et al., 2001), we hypothesized that the reduction observed in the OE thickness may result in the alterations of glomeruli morphology. To accomplish this, a quantitative analysis of the cross-sectional area of individual glomeruli was performed across ACE2 KO (558 glomeruli) and WT mice (496 glomeruli). The cumulative frequency distribution of the glomeruli area revealed a reduction in the cross-sectional area of olfactory glomeruli in ACE2 KO mice compared to WT, C57BL/6 J animals (Figures 2E1-E3, WT: 335.6 ± 6.444 μm^2^, ACE2 KO: 283.1 ± 5.191 μm^2^, K-S test, *p* < 0.0001). In addition to the cross-sectional area, we also analyzed the perimeter and minimum and maximum diameter. The perimeter and minimum and maximum diameter for glomeruli of the ACE2 KO animals were lower than that of the WT animals (Supplementary Figure S2). Further, to investigate the potential modifications in the neural circuits caused by the knockout of ACE2 receptors, we performed the immunostaining for the neuronal cytoskeletal protein MAP2, which stains the neurites. The qualitative analysis revealed a more prominent and discernible neurite projections in the glomeruli of WT animals. To quantify these changes, we calculated the mean gray value corresponding to MAP2 immunoreactivity in individual glomeruli of ACE2 and WT animals. A total of 558 glomeruli in ACE2 KO mice and 496 glomeruli in WT animals were analyzed and the cumulative distribution of intensities were compared across WT and ACE2 KO animals. This quantification revealed significantly lower MAP2 immunoreactivity in ACE2 KO compared to control mice implying severe alterations in neural circuits caused by the knockout of ACE2 receptors (Figures 2F1-F3, WT: 2.490 ± 0.0499, ACE2 KO: 2.357 ± 0.0517, K-S test, *p* = 0.0349). Taken together, these results prove morphological aberrations in the sensory periphery (OE) as well as in the pre-cortical sensory area (OB) of ACE2 KO mice, which may cause alterations in the sensory and cognitive abilities of animals.

### 3.3. Altered odor detection and discrimination behavior in ACE2 KO mice

**Figure 3:**
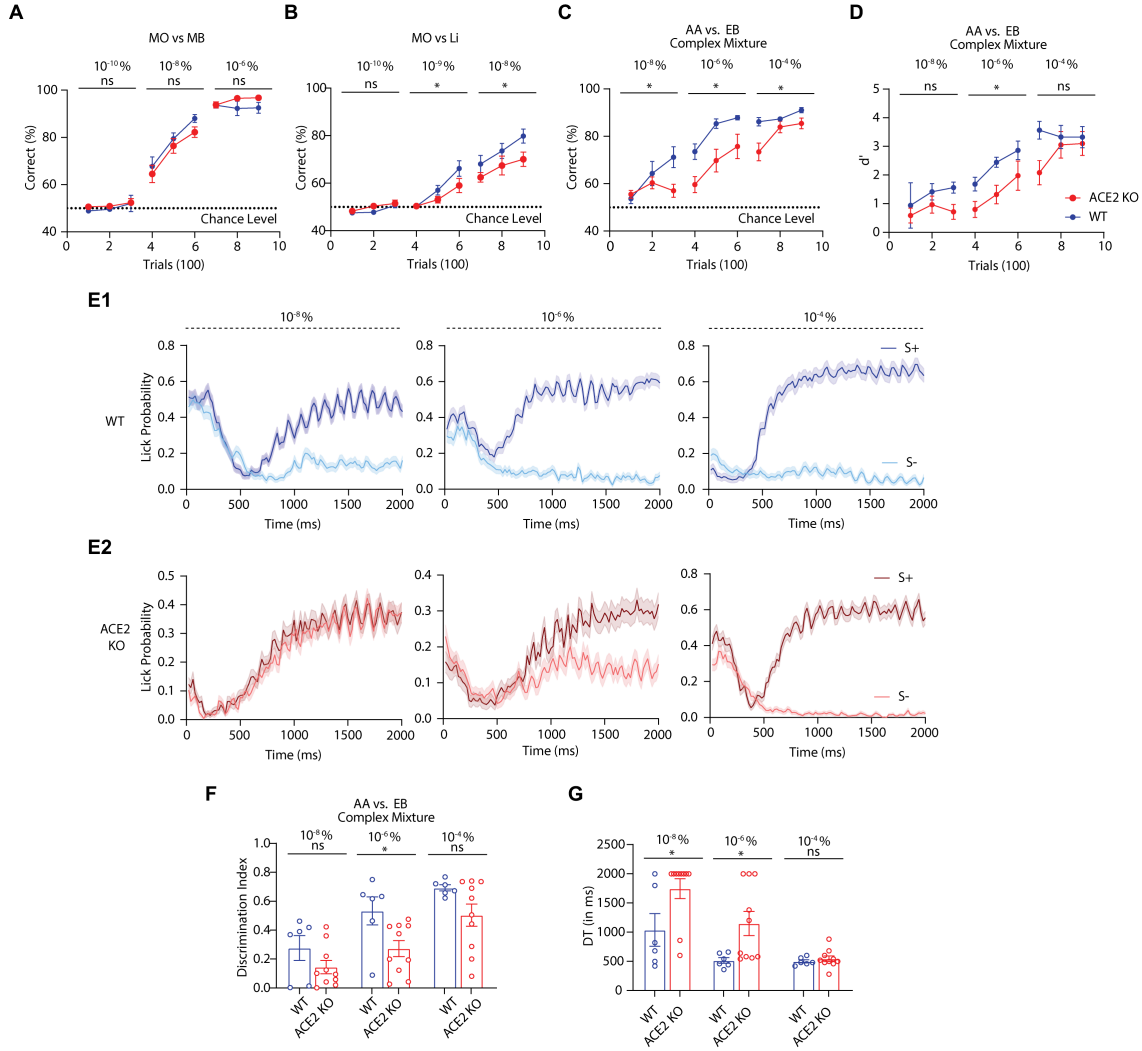
Odor detection and discrimination characteristics of WT and ACE2 animals. **(A)** Accuracy of performance shown by learning curves for WT and ACE2 KO animals when trained to discriminate mineral oil (MO) vs. Methyl Benzoate (MB) at different concentrations (10^−10^%, 10^−8^%, and 10^−6^%). Comparisons of learning curves: Two-Way ANOVA. For, 10^−10^%: [*F*(1,45) = 0.8053, *p* = 0.3743], 10^−8^%: [*F*(1,45) = 2.495, *p* = 0.1212], and 10^−6^%: [*F*(1,45) = 4.236, *p* = 0.0554], number of animals: n_ACE2KO_ = 10, n_WT_ = 7. **(B)** Learning curves of WT and ACE2 KO animals for mineral oil (MO) vs. Limonene (+) (Li) at different concentrations (10^−10^%, 10^−9^%, and 10^−8^%). Comparisons of learning curves: Two-Way ANOVA. For, 10^−10^%: [*F*(1,66) = 3.451, *p* = 0.0677], 10^−9^%: [*F*(1,63) = 4.435, *p* = 0.0392], and 10^−8^%: [*F*(1,63) = 7.574, *p* = 0.0077], number of animals: n_ACE2KO_ = 11, n_WT_ = 13. **(C)** Learning curves of WT and ACE2 KO for a complex odor discrimination task [60% Amyl acetate (AA) + 40% Ethyl Butyrate (EB) vs. 60% EB + 40% AA, at different concentrations (10^−8^%, 10^−6^%, and 10^−4^%)]. Comparisons of learning curves: Two-Way ANOVA. For, 10^−8^%: [*F*(1,42) = 4.605, *p* = 0.0377], 10^−6^%: [*F*(1, 42) 15.09, *p* = 0.0004], and 10^−4^%: [*F*(1, 42) = 10.09, *p* = 0.0028], number of animals: n_ACE2KO_ = 10, n_WT_ = 6. **(D)** d’ (d prime) of WT and ACE2 KO animals measured during a complex odor discrimination task, same as panel C, at different concentrations (10^−8^%, 10^−6^%, and 10^−4^%). d’ were compared between WT and ACE2 KO animals using Two-Way ANOVA. For, 10^−8^%: [*F*(1,42) = 3.360, *p* = 0.0739], 10^−6^%: [*F*(1,42) = 9.663, *p* = 0.0034], and 10^−4^%: [*F* (1, 42) = 3.277, *p* = 0.0774], number of animals: n_ACE2KO_ = 10, n_WT_ = 6. **(E)** Lick patterns of WT **(E1)** and ACE2 KO **(E2)** animals for different concentrations during the complex odor discrimination used in panel **C** and **D**. Y-axis represents the lick probability as a function of time (X-axis). **(F)** Discrimination index calculated using the lick probabilities at different concentrations. Comparison using two-tailed unpaired *t*-test: For, 10^−8^%: *p* = 0.1585, 10^−6^%: *p* = 0.0235, and 10^−4^%: *p* = 0.5276. Number of animals: n_ACE2KO_ = 10, n_WT_ = 6. **(A)** Comparison of discrimination times (DT) shown by WT and ACE2 KO animals for different concentrations, using two-tailed unpaired *t*-test: For, 10^−8^%: *p* = 0.0369, 10^−6^%: *p* = 0.0367, and 10^−4^%: *p* = 0.0858. Number of animals: n_ACE2KO_ = 10, n_WT_ = 6. In the figure * indicates *p* < 0.05, ns indicates: non-significant.

Having observed the morphological aberrations in ACE2 KO mice, we next asked how these aberrations are affecting their olfactory behavioral readouts. In asymptomatic carriers and symptomatic COVID-19 patients, we have observed compromised odor detection abilities more strikingly at the threshold levels (Bhattacharjee et al., 2020; Pardasani and Abraham, 2022; Bhowmik et al., 2023). Therefore, we investigated the detection abilities of ACE2 KO and WT mice using different batches of animals by training them on a go/no-go operant conditioning paradigm using different concentrations of specific odors vs. mineral oil (MO). On training mice to discriminate Methyl Benzoate from MO, ACE2 KO mice did not show any difference in the learning pace compared to WT animals (Figure 3A; MB, 10^−10^%, 10^−8^%, and 10^−6^%, diluted in MO, two-way ANOVA for each concentration, non-significant (ns) represents *p* > 0.05). However, ACE2 KO mice showed slower learning pace for certain concentrations on training them to discriminate (+) Limonene (10^−10^%, 10^−9^%, and 10^−8^%) from MO (Figure 3B, two-way ANOVA for each concentration, * represents *p* < 0.05 and ns represents *p* > 0.05). As these alterations can be dependent on the odorants used, a detailed screening using many odor pairs and more concentrations will be required to find out the changes in odor detectabilities.

As we observed varying detection abilities with ACE2 KO, we further studied odor discriminations using a complex binary mixture of Amyl acetate (AA) and Ethyl butyrate (EB) at varying concentrations (see methods). We quantified and compared various behavioral readouts from KO and WT animals to confirm the behavioral phenotypes caused by the knockout of ACE2 receptors. KO mice showed slower learning pace compared to control animals at different concentrations (Figure 3C, two-way ANOVA for each concentration, * represents *p* < 0.05). To account for the hit and false alarm probabilities while learning the discrimination task, d-prime (d’) was calculated for both KO and WT groups and significant differences were observed (Figure 3D, two-way ANOVA for each concentration, * represents *p* < 0.05 and ns represents *p* > 0.05). Further, on analyzing the lick behavior with high temporal precision, we observed the differences in their licking responses toward rewarded and non-rewarded odors (Figures 3E1,E2, see methods). Therefore, we calculated the discrimination index based on this and found significant differences between KO and WT groups (Figure 3F, see methods, two-tailed unpaired t-test for each concentration, * represents *p* < 0.05 and ns represents *p* > 0.05). Further, the difference in the reaction times, quantified by discrimination times (DT, see methods), showed slower times for the KO compared to control mice (Figure 3G, two-tailed unpaired t-test for each concentration, * represents *p* < 0.05 and ns represents *p* > 0.05). In summary, this detailed behavioral phenotyping confirms the olfactory sensory and cognitive deficits due to the knockout of ACE2 receptors.

### 3.4. Impaired novel odor discrimination in ACE2 KO mice

**Figure 4:**
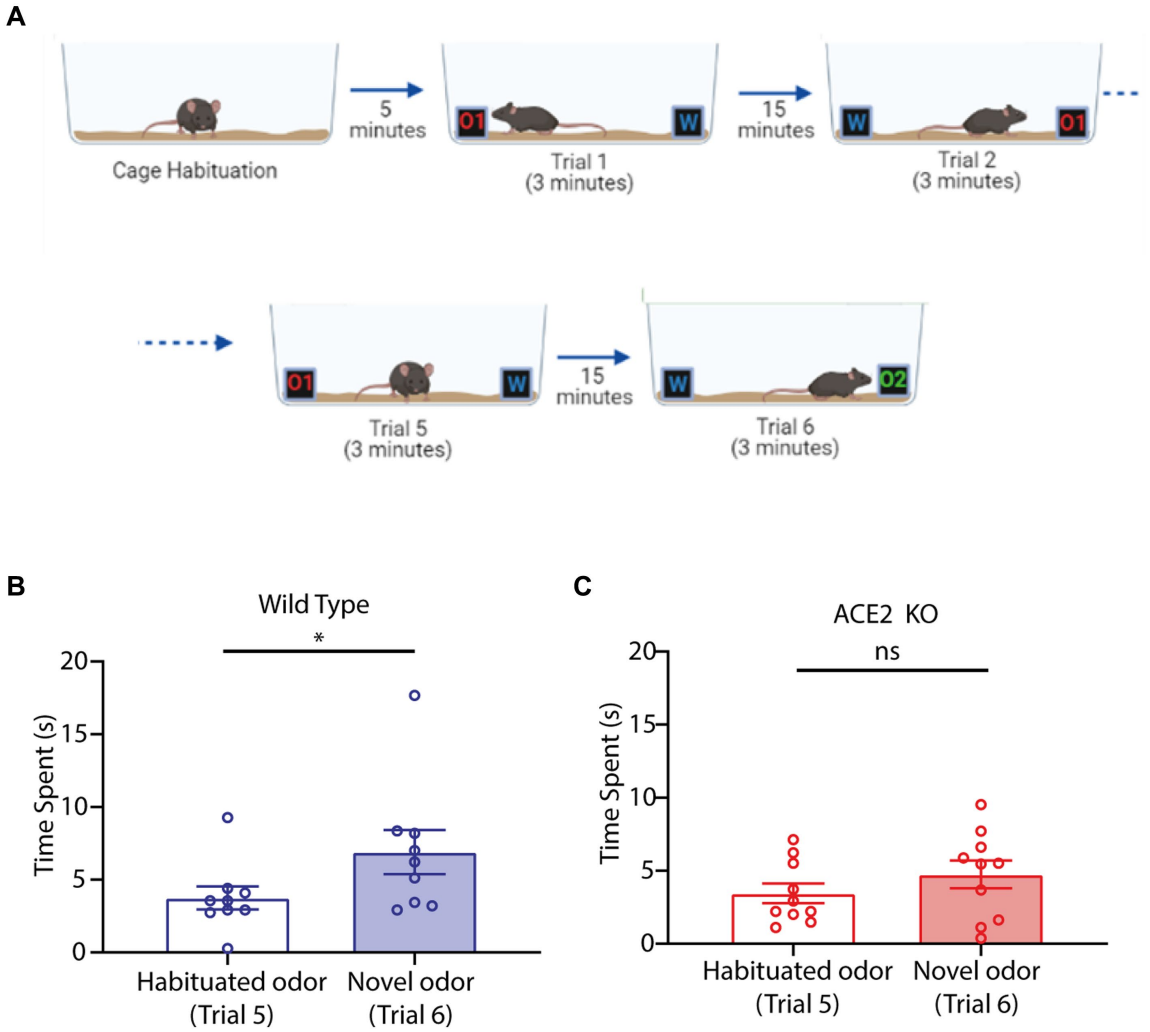
Novel odor discrimination is impaired in ACE2 KO animals. **(A)** Schematic of the novel discrimination task. The task begins with animals getting habituated with the cage followed by 5 trials of odor habituation wherein at one end an odorant (O1) was provided whereas on the other end there was water (W). For each trial the cage was rotated by 180° to mitigate any non-specific preference. Following habituation, the odor O1 was replaced by a novel odor (O2) and time spent by animals near the novel odor (Trial 6) vs. habituated odor (Trial 5) was used to assess the novel odor discrimination ability. **(B)** Comparison of time spent by WT animals during the task near the habituated and the novel odor. Time spent by animals near habituated odor (3.753 ± 0.7959 s) was significantly lower than that for novel odor (6.908 ± 1.514 s), one-tailed paired *t*-test, *p* = 0.0132, *n* = 9. **(C)** Comparison of time spent by ACE2 KO animals during the task near the habituated and the novel odor. Time spent by animals near habituated odor (3.449 ± 0.6708 s) and novel odor (4.743 ± 0.9469 s) was similar, one-tailed paired t-test, *p* = 0.0965, *n* = 10.

Olfactory dysfunctions are reported as early symptoms in Parkinson’s disease (PD) patients (Barresi et al., 2012; Doty, 2012; Fullard et al., 2017; Marin et al., 2018). In addition, a few cases of PD associated with COVID-19 infection have been reported (Li et al., 2020; Sulzer et al., 2020; Merello et al., 2021). As olfactory deficits and PD are strongly linked, we decided to test ACE2 KO mice on a habituation and novel odor discrimination task that is used to phenotype PD mouse models (Fleming et al., 2008; Lehmkuhl et al., 2014). Animals’ ability to discriminate between a familiar odor and a novel odor was assessed by quantifying the time spent by them to sample the new odor after getting exposed to another stimulus (familiar odor) few times. In brief, mice were exposed to an odor for 3 min on one side of a cage while the opposite side had similar box without any odor. They were exposed five times rotating the cage 180 degrees for each trial (Figure 4A). The novel odor discrimination was assessed by comparing the time spent by animals in sampling the novel odor vs. the habituated odor. While WT mice spent a significantly longer time for exploring the novel odor compared to the habituated odor (Figure 4B, Habituated odor: 3.753 ± 0.7959 s, Novel odor: 6.908 ± 1.514 s, one-tailed paired *t*-test, *p* = 0.0132), ACE2 KO animals spent a similar amount of time exploring both the odors (Figure 4C, Habituated odor: 3.449 ± 0.6708 s, Novel odor: 4.743 ± 0.9469 s, one-tailed paired *t*-test, *p* = 0.0965). Hence, our results revealed impairments in novel olfactory discriminations in animals with knockout of ACE2 receptors.

### 3.5. ACE2 KO female mice display compromised multimodal pheromonal location memory

**Figure 5:**
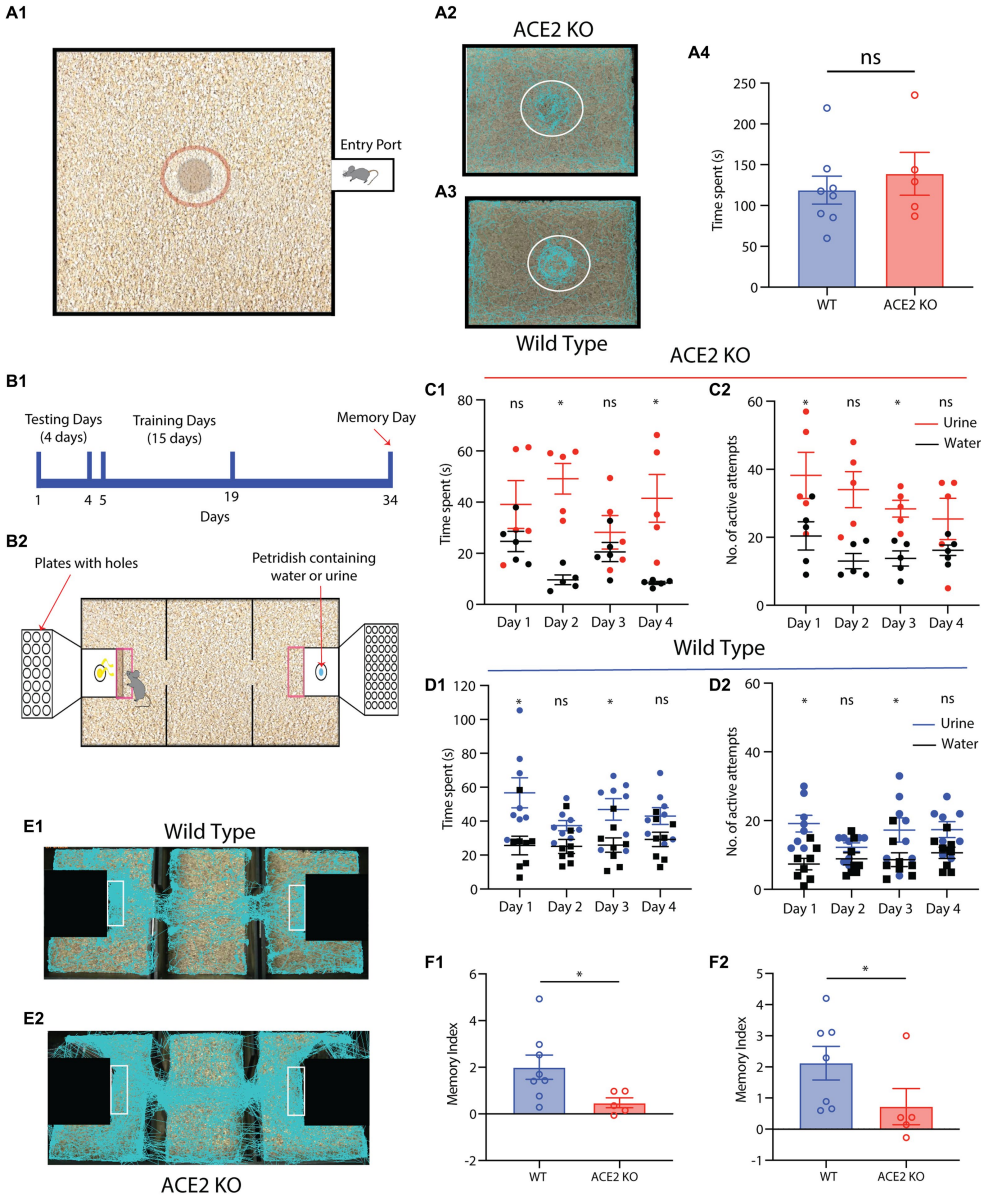
ACE2 KO animals show impaired memory in a multimodal pheromone learning task. **(A)** Pheromone detection abilities of ACE2 KO animals are similar to that of the WT animals. **(A1)** Diagrammatic representation of the setup used for pheromonal detection assay. The dimensions of the setup are 60 cm x 45 cm. A petri dish containing male soiled bedding and urine was placed at the center of the arena and females were introduced in this arena. The animals were tracked using EthoVision software while their motions were captured on camera. The time spent by females near to the petri dish was measured in order to gage their pheromonal detection abilities. **(A2,A3)** Representative tracks taken by ACE2 KO and WT animals during the pheromone detection task, respectively. **(A4)** The pheromonal detection abilities of both groups of the animals was similar (WT: 118.8 ± 17.06 s, ACE2 KO: 138.8 ± 26.19 s, two-tailed unpaired *t*-test, *p* = 0.5143, n_ACE2KO_ = 5, n_WT_ = 8). **(B) (B1)** Timeline of multimodal pheromone location learning task. Animals undergo testing for first 4 days, followed by 15 days training. Fifteen days post completion of the training, the memory of the animals was assessed. **(B2)** Illustration of the setup used for training the animals to associate the urine smell and neutral stimuli with specific orifice diameters. **(C,D)** Sampling parameters of ACE2 KO and Wildtype (WT) animals during first 4 days of testing, respectively. **(C1)** Time spent by ACE2 KO females near the water and urine zone during the testing days (two-way ANOVA with Bonferroni’s multiple comparison test, * represents *p* < 0.05 and ns represents *p* > 0.05). **(C2)** Number of active attempts by ACE2 KO females near the water and urine zone during the testing days (two-way ANOVA with Bonferroni’s multiple comparison test, * represents *p* < 0.05 and ns represents *p* > 0.05, n_ACE2KO_ = 5). **(D) (D1)** Time spent by WT females near the water and urine zone during the testing days (two-way ANOVA with Bonferroni’s multiple comparison test, * represents *p* < 0.05 and ns represents *p* > 0.05). **(D2)** Number of active attempts by WT females near the water and urine zone during the testing days (two-way ANOVA with Bonferroni’s multiple comparison test, * represents *p* < 0.05 and ns represents *p* > 0.05, n_WT_ = 8). **(E) (E1,E2)** Representative tracks during the memory day for WT and ACE2 KO animals, respectively. **(F)** Comparison of Memory index between ACE2 KO and WT animals calculated using time spent and the number of active attempts. **(F1)** ACE2 KO females showed impaired memory (index calculated using time spent) compared to the WT animals (WT: 2.002 ± 0.5185, ACE2 KO: 0.4774 ± 0.2123, two-tailed unpaired *t*-test, *p* = 0.0483). **(F2)** ACE2 KO females showed impaired memory (index calculated using number of active attempts) compared to the WT animals (2.118 ± 0.54, ACE2 KO: 0.7249 ± 0.5810, two-tailed Mann–Whitney test (non-normal distribution), *p* = 0.0186, n_ACE2KO_ = 5, n_WT_ = 7–8).

Rodent olfactory subsystems can process various types stimuli including pheromones. Volatile components of pheromones have been shown to be processed by main olfactory bulb (MOB) (Buck, 2000). As we observed morphological aberrations in the MOB, we studied the pheromone detection abilities of ACE2 KO mice. When presented with pheromonal cues from the opposite sex in form of the soiled bedding, sexually mature ACE2 KOs and control female mice explored these cues in a similar manner. This was quantified by measuring the time spent near to the soiled bedding kept at the center of an open arena (Figures 5A1-A4, WT: 118.8 ± 17.06 s, ACE2 KO: 138.8 ± 26.19 s, two-tailed unpaired t-test, *p* = 0.5143). This result shows similar pheromonal detection abilities in ACE2 KO and control animals.

In nature, pheromone location information helps animals finding their mates and avoiding potential predators. This information can also be carried by the substances where the semiochemicals are being sprayed. Therefore, involvement of whiskers along with the olfactory system is anticipated in enabling the multimodal association between pheromones and their locations. Hence, we investigated whether ACE2-KO mice show any deficits in the acquisition of this multimodal information. We employed an established behavioral paradigm to quantify the multimodal learning of pheromonal locations (Pardasani et al., 2021). When mice were allowed to explore both pheromone and neutral stimulus containing chambers (which were closed with lids having holes of different diameters, see Materials and Methods for the details, Figures 5B1,B2) in a three-chambered assay, no consistent preferences toward either of these chambers were observed. Both groups spent time in front of the chamber and displayed active attempts in sampling pheromonal cues, confirming their detection abilities (Figures 5C1-D2, two-way ANOVA with Bonferroni’s multiple comparison test for time spent and number of active attempts for both groups of animals, * represents *p* < 0.05 and ns represents *p* > 0.05). Both groups of mice were then trained for 15 days to associate pheromonal cues with varying orifice’ diameters on the lid of the respective chambers (see methods). Their memory of multimodal association was assessed 15 days after the completion of training. The time spent in front of the urine chamber and the number of active attempts for sampling pheromonal cues were used to calculate the corresponding memory index. ACE2 KO females exhibited significantly lower memory index compared to the WT animals (Figures 5E1-F2, Memory index; Time Spent, WT: 2.002 ± 0.5185, ACE2 KO: 0.4774 ± 0.2123, two-tailed unpaired *t*-test, *p* = 0.0483; Number of active attempts, WT: 2.118 ± 0.54, ACE2 KO: 0.7249 ± 0.5810, two-tailed Mann–Whitney test, *p* = 0.0186). These results imply impaired cognitive abilities caused by the knockout of ACE2 receptors.

### 3.6. ACE2 KO mice did not display any anxiety or depression phenotypes

**Figure 6:**
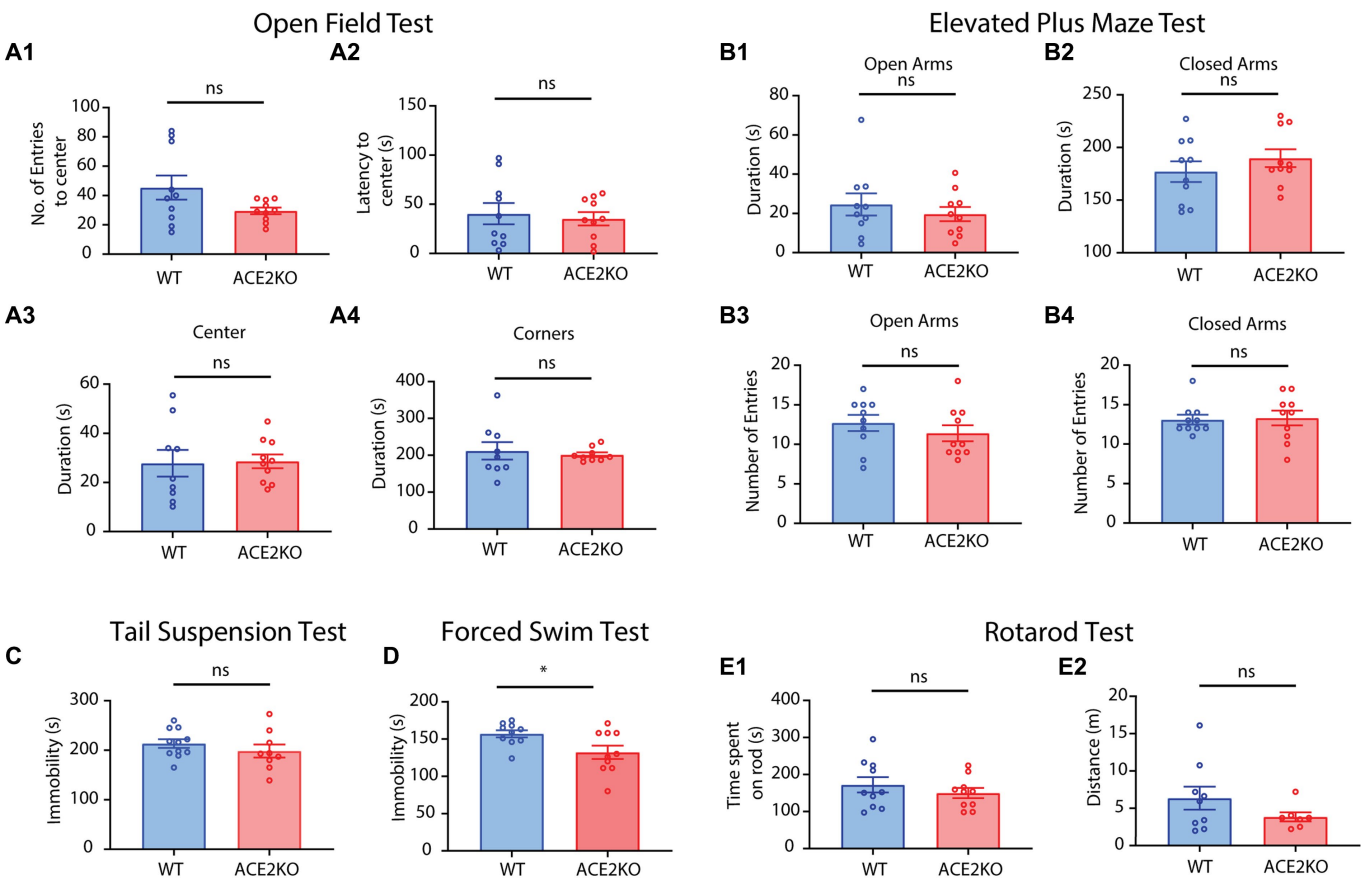
ACE2 KO and WT animals showed no differences in mood disorder-related behaviors. **(A)** Comparison of different parameters between ACE2 KO and WT animals for an open field test (OFT). **(A1)** Number of entries to the center between WT (45.40 ± 8.224) and ACE2 KO (29.50 ± 2.296) animals was similar (two-tailed unpaired *t*-test, *p* = 0.0790). **(A2)** Latency to the center between WT (40.34 ± 10.83 s) and ACE2 KO (35.17 ± 6.170) animals was similar (two-tailed unpaired *t*-test, *p* = 0.6895). **(A3)** Time spent in the center between WT (27.81 ± 5.448 s) and ACE2 KO (28.60 ± 2.825) animals was similar (two-tailed unpaired *t*-test, *p* = 0.8953). **(A4)** Time spent in the corners between WT (212.0 ± 23.80 s) and ACE2 KO (201.5 ± 6.158 s) animals was similar (two-tailed unpaired *t*-test, *p* = 0.6774). Number of animals: n_ACE2KO_ = 10, n_WT_ = 9–10. **(B)** Comparison of different parameters between ACE2 KO and WT animals for an elevated plus maze test (EPM). **(B1)** Time spent in open arms between WT (24.56 ± 5.666 s) and ACE2 KO (19.65 ± 3.615) animals was similar (two-tailed unpaired *t*-test, *p* = 0.4747). **(B2)** Time spent in open arms between WT (177.1 ± 9.792 s) and ACE2 KO (189.8 ± 8.491) animals was similar (two-tailed unpaired *t*-test, *p* = 0.3403). **(B3)** Number of entries in open arms between WT (12.70 ± 1.023) and ACE2 KO (11.40 ± 1.013) animals was similar (two-tailed unpaired *t*-test, *p* = 0.3784). **(B4)** Number of entries in open arms between WT (13.10 ± 0.6227) and ACE2 KO (13.30 ± 0.9434) animals was similar (two-tailed unpaired *t*-test, *p* = 0.8615). Number of animals: n_ACE2KO_ = 10, n_WT_ = 10. **(C)** Comparison of time immobile by ACE2 KO and WT animals for tail suspension test (TST). There was no significant difference in the time spent immobile between the groups (WT: 213.5 ± 8.628 s, ACE2 KO: 198.4 ± 13.30 s, two-tailed unpaired *t*-test, *p* = 0.3374, number of animals: n_ACE2KO_ = 9, n_WT_ = 11). **(D)** Comparison of time spent immobile by ACE2 KO and WT animals for forced swim test (FST). Time immobile by the ACE2 KO animals was significantly higher than that by the WT WT: 0.157.0 ± 4.798 s, ACE2 KO: 132.3 ± 9.017 s, two-tailed unpaired *t*-test, *p* = 0.0264, number of animals: n_ACE2KO_ = 10, n_WT_ = 10. **(E)** Comparison of different parameters between ACE2 KO and WT animals for rotarod test. **(E1)** There was no significant difference in the time spent on the rod between the groups (WT: 172.2 ± 20.61 s, ACE2 KO: 149.9 ± 13.55 s, two-tailed unpaired *t*-test, *p* = 0.3762). **(E2)** There was no significant difference in the total distance covered between the groups (WT: 6.356 ± 1.544 m, ACE2 KO: 3.836 ± 0.6166 m, two-tailed unpaired *t*-test, *p* = 0.1935).

Having observed sensory as well as cognitive deficits in ACE2 KO mice, we further studied if these phenotypes were triggered by any anxiety-related or depressive behaviors that might be caused by the knockout of ACE2 receptors. This study was also prompted by the observations of long-term sensory and cognitive deficits (Bhattacharjee et al., 2020; Bhowmik et al., 2023) and mood disorders (Lamontagne et al., 2021) reported during and post-COVID conditions. We employed an array of commonly used behavioral paradigms to quantify these behaviors (Belovicova et al., 2017). On conducting open field test with experimental and control groups of mice, we did not observe any differences between the groups in the number and latency of entries to the center and the time spent in the center and the corners of the field (Figures 6A1-A4, two-tailed unpaired t-test for each parameter, ns represents *p* > 0.05). In the elevated plus, we did not observe any differences in the time spent and number of entries in the open and closed arms (Figures 6B1-B4, two-tailed unpaired t-test for each parameter, ns represents *p* > 0.05). These results indicate the absence of any anxiety-related behaviors due the knockout of ACE2 receptors. To test for any depressive phenotypes, we conducted tail suspension and forced swim tests. Time of immobility was not different between the control and knockout groups of mice in the tail suspension test (Figure 6C, two-tailed unpaired *t*-test, *p* = 0.3374). In the forced swim test, immobility was slightly higher for the WT group, indicating the absence of any depressive symptoms in ACE2 KO mice (Figure 6D, two-tailed unpaired t-test, *p* = 0.0264). Further, we carried out the rotarod test to see if there are any motor deficits in ACE2 KO mice and time spent on the rotarod and the distance covered by both groups of animals were found to be similar (Figures 6E1,E2, two-tailed unpaired *t*-test, ns represents *p* > 0.05), indicating the absence of any motor dysfunctions due to the knockout of ACE2 receptors. All these tests provide the evidence for the absence of any mood disorder-related phenotypes in ACE2 knockout mice. Taken together, our results prove sensory and cognitive deficits in ACE2 KO mouse model, supported by the morphological aberrations we observed in these mice.

## 4. Discussion

Neurological complications of long-COVID may challenge the global health for many more years (Kay, 2022). Various olfactory problems including hyposmia, anosmia, and parosmia have been reported during infection and under long-COVID conditions (Bhattacharjee et al., 2020; Pardasani and Abraham, 2022; Bhowmik et al., 2023). While infection at the olfactory periphery and the neuronal loss may explain the transient hyposmic and anosmic conditions, parosmia may result from the mis-targeting of regenerating OSNs during the recovery period (Costanzo, 2000; John and Key, 2003; Cooper et al., 2020). The expression of ACE2 receptors, that mediates virus infection in sustentacular cells of olfactory epithelium, explains the severe olfactory problems under COVID-19 infection. Therefore, we aimed to generate a complete ACE2 knockout mouse model using the CRISPR-Cas9 based genome editing method and investigate its function in modulating olfactory information processing. The deletion of ACE2 receptors may not lead to all pathophysiological conditions caused by SARS-CoV2 infection. However, the loss of ACE2 receptor function in the supporting sustentacular cells may ultimately result in the ionic imbalance in the epithelium, causing the cell death of olfactory sensory neurons, hence leading to various olfactory dysfunctions (Cooper et al., 2020).

Transgenic models can be created by different approaches. For example, in Cre-Lox recombination system, the expression specificity is achieved by crossing floxed mouse lines with Cre driver lines or by delivering Cre recombinase in a cell type-specific manner (Capecchi, 2005; Taniguchi et al., 2011). The generation of these mouse lines takes longer than a year as it requires extensive backcrossing to screen for a homogenous background. The recent advancement in the CRISPR-Cas9 genome editing tools have enabled the researchers to generate robust mouse models with targeted genetic background comparatively faster (Nishizono et al., 2020). The knockouts and knockins can be directly generated by injecting the guide RNA and Cas9 into the pronucleus of fertilized mouse eggs (Yang et al., 2013). The existing ACE2 knockout mouse models created with CRISPR-Cas9 were mostly used to study the pulmonary and cardiovascular systems, and differences in knockout phenotypes are being reported. The genetic make-up of the models may attribute to the inconsistencies of the observed phenotypes, which can be considered as a drawback of the CRISPR-Cas9 approach (Jia et al., 2020). In this study, the deletion of ACE2 gene was ensured by targeting the crucial translational start site of the exon 2 and was confirmed by sequencing and western blotting.

The COVID-19 pandemic struck the world recording a high mortality rate and causing a decrease of human wellbeing globally. Since the start of the pandemic, numerous studies have been conducted to identify the various entry routes of SARS-CoV-2. The human angiotensin-converting enzyme 2 (hACE2) receptor was confirmed to be the target of the spike glycoprotein of the virus (Whittaker et al., 2021). After the viral glycoprotein binds to the ACE2 receptor, the TMPRSS2 protein cleaves the virus’ S2 site, causing the internalization of the virus (Glowacka et al., 2011; Jackson et al., 2022). The widespread expression of ACE2 receptors indicates toward number of possible entry points for the invasion of virus (Mainland et al., 2015; Sungnak et al., 2020; Zhou et al., 2020; Boldrini et al., 2021; Casagrande et al., 2021; Huang N. et al., 2021; Pardasani and Abraham, 2022). Additionally, the virus can also enter the body through a breach of the blood–brain barrier (BBB), which is caused by the instability of the barrier by an increase in inflammatory cytokines following infection (Huang X. et al., 2021). Despite the debate over the virus’s route of entry, it is recognized to be associated to the ACE2 receptors. As SARS-CoV2 entry into the cells through membrane fusion is thought to down-regulate the ACE2 receptors with a loss of these receptors’ catalytic effect (Verdecchia et al., 2020), we created the ACE2 KO mouse model to mimic the effects of COVID-19 and studied its long-term effects. Even though it may not alter organ systems’ functions as during or after COVID-19 infection, the ACE2 KO model offers a platform for the exploration of various parameters that can affect overall human well-being as a result of long-COVID-19.

The COVID-19 infection leads to morphological and functional alterations in the brain (Douaud et al., 2022; Du et al., 2023). Since the sustentacular cells of the olfactory epithelium contain the ACE2 receptor, we started by examining the impact of ACE2 deletion on the morphology of the olfactory epithelium (Brann et al., 2020). The width of the epithelium was considerably smaller in the ACE2 KO animals than in the control animals. Additionally, the glomeruli’s cross-sectional area and MAP2 immunoreactivity were both reduced in the ACE2 KO animals. These findings demonstrate the changes brought about by the deletion of ACE2 in the olfactory epithelium and olfactory bulb. In humans, COVID-19 has been shown to increase apoptosis and decrease neurogenesis in the hippocampus (Bayat et al., 2022). In mouse models, it has been shown that ACE2 loss causes a reduction in the exercise-induced hippocampal neurogenesis (Klempin et al., 2018; Alenina and Bader, 2019). Given the relationship between ACE2 and COVID-19 and the fact that the olfactory epithelium is another part of the brain where neurogenesis occurs (Crews and Hunter, 1994), it is probable that the decreased width of the OE is the result of increased apoptosis or reduced neurogenesis. Further studies investigating apoptosis and OSN turnover would be needed to confirm this.

The COVID-19 infection also causes learning and cognitive impairments that even persisted in the post-COVID conditions (Hampshire et al., 2021; Hugon et al., 2022; Bhowmik et al., 2023). Our observations of ACE2 KO animals having deficits in detection, discrimination, and novel odor recognition were similar to clinical observations made in patients. Humans’ orbitofrontal cortex has extensive connections to other cortical areas and may help in processing of complex olfactory inputs (McGann, 2017). A decrease in the thickness of gray matter in the orbitofrontal cortex as a consequence of the COVID-19 may also contribute to severe olfactory and cognitive dysfunctions observed in humans (Douaud et al., 2022). In support of these clinical observations, earlier ACE2 KO mouse models exhibited learning impairments in Morris water maze and Y-maze tasks (Wang et al., 2016). In addition, ACE2 activation in the brain has been proven to have protective effects against the cognitive decline caused by amyloid pathology in a mouse model of Alzheimer’s Disease (Evans et al., 2020). Despite these supporting evidence, the effect of CRISPR-Cas9 based disruption of ACE2 expression on other gene networks, may not mimic the exact pathophysiological conditions caused by COVID-19 (Stephan et al., 2022).

Rodents’ olfaction is critical for their social and reproductive behaviors. During courtship behavior, olfactory system detects pheromones and recognizes their location (Pardasani et al., 2021). Here, we also investigated how ACE2 KO affected the animals’ capacity for pheromone detection and the association of pheromones with their location. While the pheromonal detection abilities were unaffected, the ACE2 KO animals displayed poor memory of the pheromone location association, implying the impact of ACE2 deletion on rodents’ social and reproductive behaviors. As a result of COVID-19 pandemic, a decline in sexual interest, and frequency were observed (Pascoal et al., 2021). In contrast, a few populations showed an increase in sexual desire, however with a reluctance toward conception (Yuksel and Ozgor, 2020). These findings emphasize the negative impact of the pandemic on human sexual health and success. These problems with reproductive health might be transient and may have resulted by COVID-19’s detrimental effects on mental health. According to World Health Organization’s assessment, the pandemic caused a 25% rise in the prevalence of anxiety and depression globally (WHO, 2022). However, over time, the behaviors linked to the deterioration of mood disorders such as anxiety and depression was diminishing, suggesting that these effects are transient and may have been attributed to a variety of factors during the pandemic (Manchia et al., 2022). In our analysis of mood disorder related behaviors, ACE2 KO animals did not show any depression or anxiety phenotypes.

In summary, our results demonstrate that knockout of ACE2 receptors leads to sensory and cognitive disabilities, which were similar to clinical observations made from COVID-19 patients. Further, our experimental strategy provides a potential method for probing the neural mechanisms of cognitive deficits under long COVID conditions.

## Data availability statement

The original contributions presented in the study are included in the article/Supplementary material, further inquiries can be directed to the corresponding authors.

## Ethics statement

The animal study was reviewed and approved by Institutional Animal Ethics Committee (IAEC), IISER Pune.

## Author contributions

NA and SG conceptualized the study. NA supervised all aspects of behavioral phenotyping and morphological analysis. SG supervised all aspects of knockout generation. NA carried out the experimental design. SM, DS, and AS performed behavioral, immunohistochemistry and microscopy experiments and analyzed the data. PS, SDM, and KS performed behavioral experiments and analyzed the data. MS performed knockout generation. SDM performed western blotting experiments. NA and SM wrote the manuscript with comments from others. All authors contributed to the article and approved the submitted version.

## Funding

This work was supported by the DBT/Wellcome Trust India Alliance intermediate grant (IA/I/14/1/501306 to NA), DST-Cognitive Science Research Initiative, Government of India (DST/CSRI/2017/271 to NA), JC Bose Fellowship from the Science and Engineering Research Board, Government of India (JCB/2019/000013 to SG), CSIR Fellowship, Government of India (SM and SDM) and UGC Fellowship, Government of India (KS) Part of the work was carried at the National Facility for Gene Function in Health and Disease (NFGFHD) at IISER Pune, supported by a grant from the Department of Biotechnology, Government of India (BT/INF/22/SP17358/2016).

## Conflict of interest

The authors declare that the research was conducted in the absence of any commercial or financial relationships that could be construed as a potential conflict of interest.

## Publisher’s note

All claims expressed in this article are solely those of the authors and do not necessarily represent those of their affiliated organizations, or those of the publisher, the editors and the reviewers. Any product that may be evaluated in this article, or claim that may be made by its manufacturer, is not guaranteed or endorsed by the publisher.

## Footnotes

^1^http://crispor.tefor.net/
