# Supplementary material for: Knockout of angiotensin converting enzyme-2 receptor leads to morphological aberrations in rodent olfactory centers and dysfunctions associated with sense of smell
Source: Front Neurosci. 2023 Jun 19;17:1180868. doi: 10.3389/fnins.2023.1180868 (PMC10315482; doi:10.3389/fnins.2023.1180868)
Supplement: Supplementary file 1 [file Data_Sheet_1.pdf]

Supplementary Figures

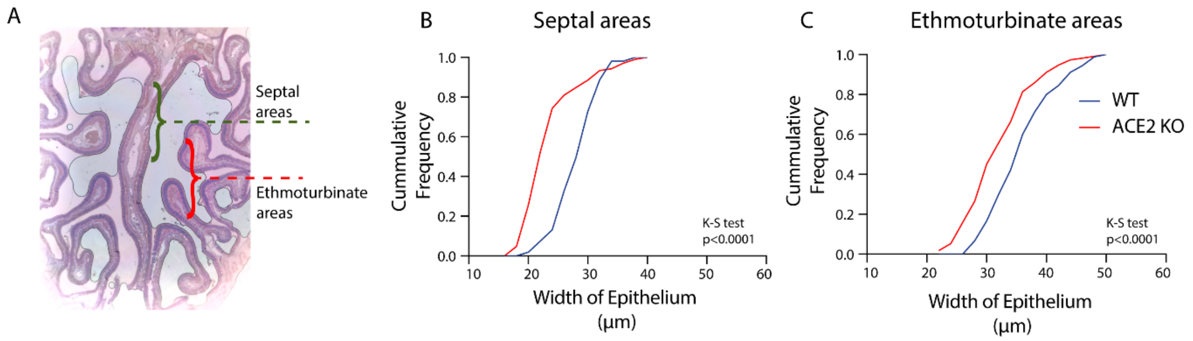

Supplementary Figure S1. Reduced olfactory epithelium width in ACE2 KO animals at different turbinate locations. Cumulative frequency distribution of width of OE for WT and ACE2 KO animals in (B) septal areas (WT: 28.73 ± 0.3367 µm; ACE2 KO: 24.11 ± 0.4647, K-S test, $p < 0.0001$) and (C) ethmoid turbinate areas (WT: 36.52 ± 0.5797; ACE2 KO: 32.72 ± 0.5156, K-S test, $p < 0.0001$).

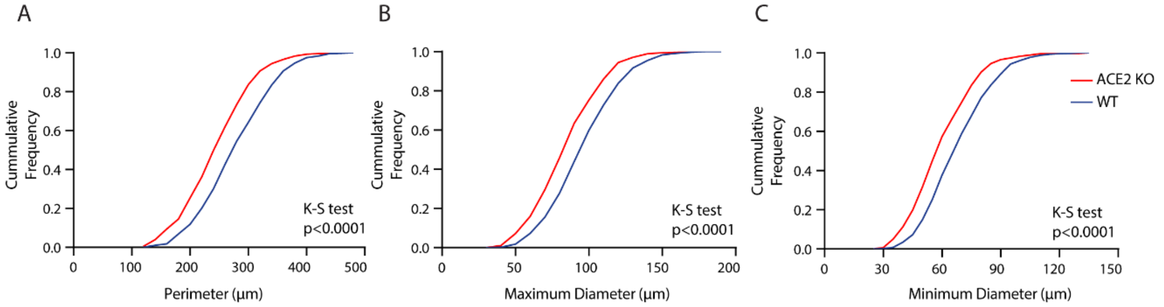

Supplementary Figure S2. Cumulative frequency distribution of A) Perimeter (WT: 285.9 ± 2.841 µm; ACE2 KO: 252.3 ± 2.533, K-S test, $p < 0.0001$), B) Maximum Diameter (WT: 100.4 ± 1.097 µm; ACE2 KO: 88.64 ± 0.9765, K-S test, $p < 0.0001$), and C) Minimum diameter of glomeruli in ACE2 KO and WT animals. (WT: 69.96 ± 0.7613 µm; ACE2 KO: 61.56 ± 0.6862, K-S test, $p < 0.0001$).
